# Endovascular Embolization of an Aberrant Bronchial Artery Originating from the Internal Mammary Artery in a Patient with Hemoptysis

**DOI:** 10.1155/2016/2707195

**Published:** 2016-06-07

**Authors:** Hiroyuki Fujii, Akifumi Fujita, Hiroyasu Nakamura, Takahiro Sasaki, Hideharu Sugimoto

**Affiliations:** Department of Radiology, Jichi Medical University, School of Medicine, 3311-1 Yakushiji, Shimotsuke, Tochigi 329-0498, Japan

## Abstract

Massive hemoptysis is a life threatening respiratory emergency with high mortality and the bronchial artery (BA) is its most frequent source. Herein, we report a case of a 76-year-old man with recurrent hemoptysis due to an aberrant right BA arising from the right internal mammary artery (IMA), an extremely rare origin, that was clearly depicted on pretreatment computed tomography angiography (CTA). The patient was treated successfully by transcatheter bronchial artery embolization (BAE) of the aberrant BA and the hemoptysis has since been controlled for 9 months. Knowledge of the detailed BA anatomy is essential for performing BAE, especially in cases of aberrant BA. CTA is a promising tool for pretreatment planning of emergency BAE in patients with hemoptysis.

## 1. Introduction

Massive hemoptysis is a life threatening respiratory emergency with high mortality and the bronchial artery (BA) is the most frequent source [1]. Bronchial artery embolization (BAE) is a safe and effective treatment for controlling hemoptysis [2]. A thorough knowledge of BA anatomy is essential for planning and performing BAE. However, it is important to note that the BA has many anatomical variations. For example, a BA originating outside the descending thoracic aorta between the fifth and sixth thoracic vertebrae (T5-T6) is considered as aberrant. When performing BAE for hemoptysis, undetected aberrant BAs can result in failed embolization. Recent advances in computed tomography (CT) technology have enabled detailed preoperative visualization of BA anatomy, including the presence of aberrant BAs. Herein, we presented a rare case of an aberrant right BA, arising from the right internal mammary artery (IMA), which was detected preoperatively on CT angiography (CTA) and was successfully embolized for treatment of recurrent hemoptysis.

## 2. Case Report

**Figure 1:**
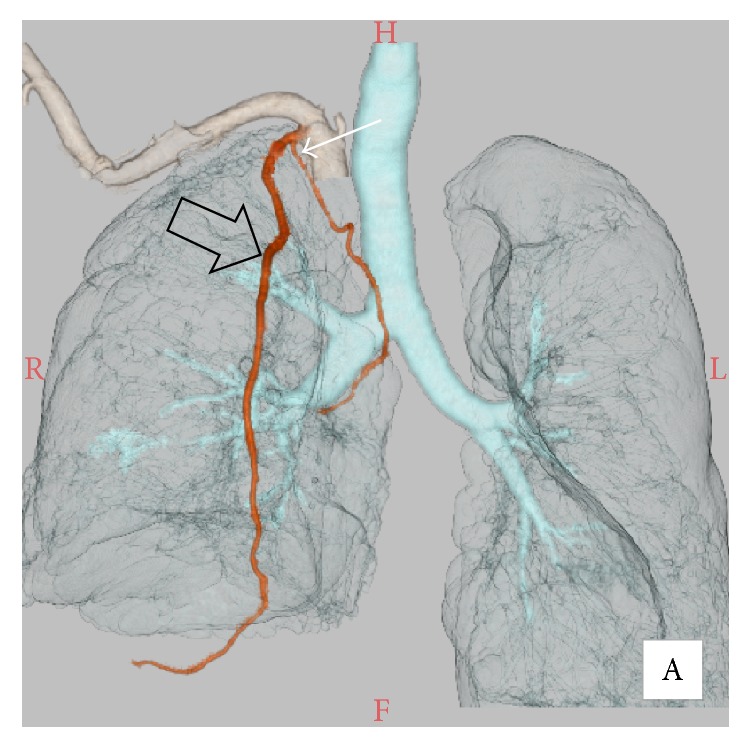
Volume rendering reconstructed image of computed tomography angiography shows a hypertrophic, aberrant right bronchial artery (white arrow) arising from the proximal portion of the right internal mammary artery (black arrow).

A 76-year-old man was admitted to our institution for endovascular embolization as management of recurrent hemoptysis. He had a history of chronic obstructive pulmonary disease. He had been taking aspirin 81 mg/day for three years after percutaneous coronary stenting for coronary artery disease. Since the patient has not undergone prior BAE, he underwent pretreatment evaluation by contrast-enhanced CT with a 64-detector row scanner (Aquilion, Toshiba, Otawara, Japan), with the arterial phase at 30 seconds and the delayed phase at 90 seconds after intravenous contrast administration (100 mL Iopamirone 300 mg/mL, Bayer Healthcare, Osaka, Japan) at 3 mL/s. The chest CT on lung window imaging showed centrilobular and paraseptal emphysematous changes in the upper lung zones and a large bulla with surrounding consolidation on the right lower lobe, which was considered as the cause of hemoptysis. The CTA clearly showed a hypertrophic, aberrant right BA arising from the proximal portion of the right IMA (Figure 1). There was no nonbronchial systemic artery. Since he had very tortuous brachiocephalic artery and aortic arch and because femoral artery approach seemed difficult, we planned a right radial artery approach to catheterize the right IMA.

**Figure 2:**
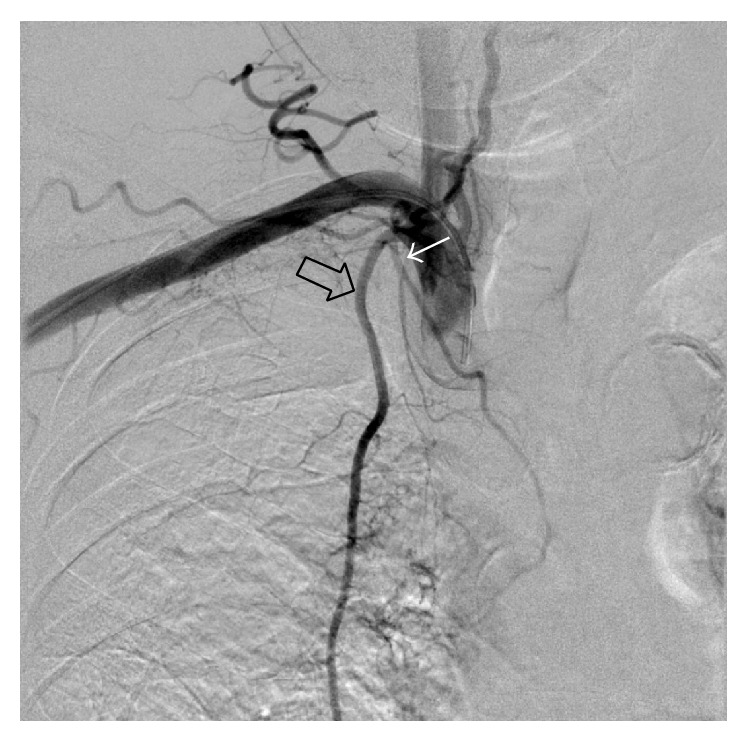
Angiogram of the brachiocephalic artery shows the hypertrophic aberrant bronchial artery (white arrow) arising from the proximal portion of the right internal mammary artery (black arrow), findings that corresponded with the CTA image.

**Figure 3:**
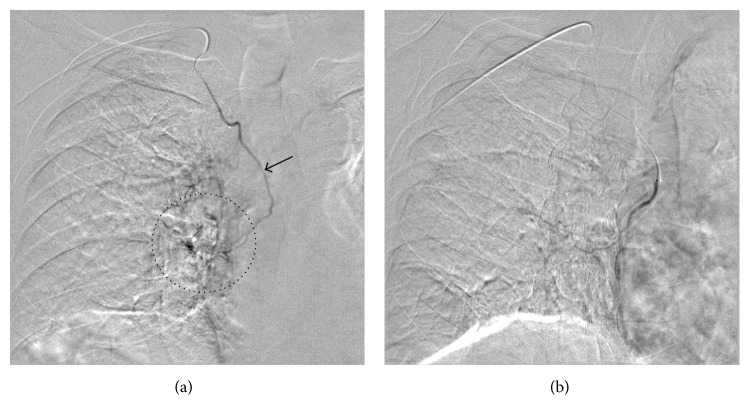
(a) Selective angiogram of the aberrant right bronchial artery (arrow) shows parenchymal staining in the right lower lobe (dotted circle). (b) The parenchymal staining disappeared after embolization with absorbable gelatin sponge.

After obtaining access through the right radial artery, selective angiogram of the brachiocephalic artery showed a hypertrophic, aberrant right BA arising from the proximal portion of the right IMA, findings that corresponded with the CTA image (Figure 2). Through the same right radial artery access, we could easily select the right IMA using a 4 Fr catheter (IMA catheter, Terumo Clinical Supply, Gifu, Japan). The aberrant right BA was successfully catheterized by a 1.9 Fr microcatheter (Progreat Σ, Terumo Clinical Supply, Gifu, Japan). Selective angiogram of the aberrant right BA showed parenchymal staining in the right lower lobe (Figure 3(a)). The microcatheter was advanced into the distal portion of the right aberrant BA, which was embolized with absorbable gelatin sponges (Spongel, Astellas Pharma Inc., Tokyo, Japan); immediately after, the parenchymal staining disappeared (Figure 3(b)). No procedure-related complications occurred and the patient experienced no further hemoptysis during a follow-up period of 9 months.

## 3. Discussion

Since the report of Remy et al. in 1973, BAE has become an established procedure in the management of massive and recurrent hemoptysis [3]. Interventional radiologists who perform BAE should have a thorough knowledge of the BA anatomy, which is highly variable in terms of origin and branching distribution. In about 70% to 83.3%, the BA most commonly arises from the descending thoracic aorta between the T5 and T6 vertebrae [4]. Cauldwell et al. classified the BA branching pattern into four classic groups based on a study on 150 adult cadavers [5]. BAs that originate outside the T5-T6 vertebral levels of the descending thoracic aorta are considered aberrant. The reported prevalence of aberrant BAs ranged from 8.3% to 35.0% [2]. An aberrant BA has been reported to originate from various arteries, such as aortic arch, brachiocephalic artery, subclavian artery, IMA, thyrocervical trunk, costocervical trunk, inferior phrenic artery, abdominal aorta, vertebral artery, or left gastric artery [6–8]. However, the likelihood of a BA arising from the IMA is extremely rare. In a study of 184 CTAs of the thoracic aorta, Battal et al. found only one BA arising from the right IMA [9]. Hartmann et al. investigated 214 patients who underwent CTA and found only three BAs arising from the ipsilateral IMA [10]. Yener et al. investigated 208 CTAs and found only one right BA originating from the right IMA and three BAs (two right BAs and one left BA) originating from the left IMA [11]. Aberrant BAs can be distinguished anatomically and angiographically from nonbronchial systemic collateral vessels in that they run along the course of the major bronchi. In contrast, nonbronchial systemic collateral vessels enter the pulmonary parenchyma through the adherent pleura or via the pulmonary ligament, and their course is not parallel to that of the bronchi [2].

Pretreatment CTA seems to play an important role in detecting an aberrant BA when planning BAE for the management of hemoptysis. Failure to recognize anatomical variations of the bronchial circulation during angiography may result in only partial control of hemoptysis, nontargeted embolization, and unsuccessful outcome [4, 10]. With recent advances in CT technology, multidetector row CT, when compared with conventional spiral CT, can now obtain two- or three-dimensional images with high quality due to shorter image acquisition time, narrower collimation, increased spatial resolution, and better isotropic data acquisition. CTA has enabled detailed evaluation of not only the aorta and its main branches, but also fine-diameter vessels, such as the BA. In the present case, we could successfully detect an aberrant right BA from the right IMA preoperatively using CTA.

We believe that the present case represented a true aberrant BA, not a nonbronchial systemic artery. There were three reasons for this. First, the patient had no prior history of BAE, which would lead to development of collateral vessels. Second, there was no other right BA arising from the aorta. Third, this artery ran along the course of the tracheobronchial tree, which is a characteristic distribution of a BA.

In conclusion, we performed BAE in a patient with an aberrant right BA from the right IMA. Pretreatment CTA is very useful for planning of BAE for hemoptysis, especially if a patient has a BA that originates from a rare source.
